# A review on the functional roles of trehalose during cryopreservation of small ruminant semen

**DOI:** 10.3389/fvets.2024.1467242

**Published:** 2024-11-19

**Authors:** Baoyu Jia, Larbi Allai, Chunyan Li, Jiachong Liang, Chunrong Lv, Guoquan Wu, Guobo Quan

**Affiliations:** ^1^College of Veterinary Medicine, Yunnan Agricultural University, Kunming, China; ^2^Yunnan Animal Science and Veterinary Institute, Kunming, Yunnan, China; ^3^Laboratory of Sustainable Agriculture Management, Higher School of Technology Sidi Bennour, Chouaib Doukkali University, El Jadida, Morocco; ^4^Higher School of Education and Training, Mohammed I University, Oujda, Morocco; ^5^Yunnan Provincial Engineering Research Center of Animal Genetic Resource Conservation and Germplasm Enhancement, Kunming, Yunnan, China; ^6^Yunnan Provincial Genebank of Livestock and Poultry Genetic Resources, Kunming, Yunnan, China

**Keywords:** semen, small ruminant, cryopreservation, trehalose, cryoinjury

## Abstract

Sperm cryopreservation is an approach to preserve sperm cells in liquid nitrogen or other cryogenic media for future use in assisted reproductive technologies, such as *in vitro* fertilization or artificial insemination. Sperm cryopreservation has been extensively used in the dairy industry and has attained excellent results after artificial insemination. However, for small ruminants the application of sperm cryopreservation is limited, due to the poor quality of frozen semen and special characteristics of the reproductive female tract. In order to improve post-thaw semen quality various cryoprotectants are used. Currently, many types of cryoprotectants, such as permeable organic solvents, sugars, antioxidants, and natural or synthetic ice blockers, have been tested on small ruminants’ sperm cryopreservation. Among them, trehalose; has shown potential acting as an excellent cryoprotectant for semen freezing. While, the exact roles and action mechanisms of trehalose during cryopreservation remain unclear. In this review, we systematically summarized the present usage status, potential action mechanisms, and future application prospects of trehalose in small-ruminant sperm cryopreservation.

## 1 Introduction

Small ruminants, primarily sheep and goats, significantly contribute to the modern livestock industry and can provide meat, wool, skin, or milk to our society. However, with the fast development of modern intensified agriculture, the genetic diversity of farm animal species is rapidly being reduced in many regions worldwide (1). Semen cryopreservation has been successfully applied to various mammalian species, including humans, domestic animals, and endangered wildlife species. Semen cryopreservation offers multiple advantages to the small ruminant livestock industry through the worldwide distribution of excellent genetic materials through artificial insemination (AI) (2, 3). It is important to take into account that the freezing and thawing process can have a harmful impact on sperm (4, 5). According to current reports, cryopreservation can lead to ice formation, cold shock, chemical effects caused by cryoprotectants, osmotic injury, oxidative injury, and apoptosis (6–8). These factors ultimately damage the structure and physiological function of sperm. Furthermore, these stresses primarily damage the plasma membrane of sperm, resulting in a significant decrease in the viability and fertility of post-thaw sperm (9, 10). In addition, the cryopreservation process causes sublethal cryoinjuries that result in approximately 50% of post-thawed sperm thus losing their viability during cervical artificial insemination with frozen–thawed semen (11).

In sheep, it has been reported that after thawing, 40 to 60% of sperm are still motile, but only 20 to 30% are biologically functional (12, 13). The use of frozen/thawed semen in conventional insemination yields poor fertility in sheep. This may be due to different factors but one of the most important restrictive reasons is considered to be the anatomical structure of the cervix (14–19). Currently, frozen–thawed semen generally attained a better results by intrauterine insemination (19–23). However, this technique needs specific equipment and well-trained technicians. Therefore, to elucidate cryoinjury mechanisms and improve the quality of frozen–thawed ram sperm still remains a great challenge until now.

The success of mammalian sperm cryopreservation is influenced by the species and individual factors (24). In addition, research and advancements in semen cryopreservation techniques continue to focus on enhancing the efficiency and success rates of sperm freezing including the optimization of the cryoprotectant solutions used, development of better freezing and thawing protocols, and application of new approaches to enhance sperm post-thaw survival and functionality (3, 25–28). Among these measures, selecting cryoprotectants is important. Cryoprotectants are substances that help protect sperm cells from damage during the freezing and thawing process (29, 30). Glycerol has been shown to have excellent cryoprotective effects on livestock semen and is now an essential component in the semen freezing extenders (31–33). Furthermore, other cryoprotectants, such as sugars (34–36), antioxidants (37–39), antifreeze proteins (40–42) and synthetic ice blockers (43, 44) have been used for semen cryopreservation. Moreover, among the sugars used in semen cryopreservation is tehalose. Previous investigations have proven that trehalose has beneficial effects on sperm during cryopreservation (45–49). Trehalose has been used for mammalian sperm cryopreservation, and previous studies have shown that it can increase sperm’s tolerance to cryoinjuries (47, 50, 51). Moreover, trehalose may have better cryoprotective effects on small ruminant sperm than other oligosaccharides, such as sucrose (48, 52, 53). However, there are still some disagreements (54, 55). More precisely, the ability of trehalose to preserve the motility of frozen sheep sperm is similar to that of sucrose (54). In another study, the effects of trehalose on frozen goat sperm are not superior to those of other oligosaccharides, including sucrose, maltose, and lactose (35). Also, it should be pointed out that trehalose, unlike glucose and fructose, is unable to pass through the plasma membrane. So, the primary function of trehalose is to protect against extracellular cryodamage (56).

The mechanisms that contribute to the cryoprotective effects of trehalose on mammalian sperm are still unclear, despite the existence of several hypotheses. The purpose of this review is to summarize the current research on trehalose for cryopreservation of small ruminant semen, which includes its current usage status, potential action mechanisms, and future application prospects.

## 2 The characteristics and potential application of trehalose

Trehalose is a typical non-reducing disaccharide composed of two glucose molecules linked together. It is naturally produced in various organisms, such as plants, fungi, bacteria, and invertebrates (57). Trehalose is known for its unique properties, which make it useful in a variety of applications. Specifically, it can act as a protective agent against stressors, such as desiccation, extreme temperatures, and oxidative damage (58). Recently, trehalose has received a lot of attention in the food and pharmaceutical industries due to its protective properties (59–61). One of the most notable characteristics of trehalose is its capacity to form a protective barrier around cells and biomolecules (57, 62, 63). Trehalose acts as a stabilizer, helping to preserve the structure and function of proteins, enzymes, and other biological molecules (61, 64). It is employed in the pharmaceutical and biotechnology industries to stabilize and maintain the active ingredients in medications, vaccines, and diagnostic kits (65). Furthermore, in the food industry, trehalose is used as a sweetener, flavor enhancer, and stabilizer for various products. Trehalose is a common ingredient in processed foods, baked goods, and beverages (66). It has the potential to enhance the texture, taste, and shelf life of these products. In addition, trehalose has been examined for its potential health benefits. According to some research, trehalose has been reported to have antioxidant and anti-inflammatory properties (67, 68), and it has the potential to be utilized in the prevention or treatment of certain diseases.

Trehalose also has the ability to act as a cryoprotectant, and is able to prevent ice crystal formation as well as maintain cell and tissue structural integrity during cryopreservation processes (47, 69–71). In the context of sperm cryopreservation, trehalose has been investigated as a potential cryoprotectant in many studies (72–75). It has been demonstrated that trehalose protects semen quality parameters from cryodamage (76–78).

Generally, the particularly cryoprotective properties of trehalose make it an intriguing substance for various applications, including sperm cryopreservation. However, it’s worth noting that other cryoprotectants are also commonly used in combination with trehalose or as alternatives, and the choice of cryoprotectants depends on the specific requirements of the cryopreservation protocol. The most effective and standardized protocols for sperm cryopreservation using trehalose as an important cryoprotectant are still being researched and optimized.

## 3 The application of trehalose in semen cryopreservation of small ruminant

The effects of trehalose on sheep (31, 49, 56, 79, 80) and goat sperm (81–84) cryopreservation have been evaluated. Most researchers support the positive effects of trehalose on sperm during the cryopreservation process. For example, the best results were obtained in sheep semen using tris, citric acid, fructose, egg yolk, glycerol supplemented with 50 or 100 mOsm of trehalose, while the post-thaw sperm quality significantly decreased with 200 and 400 mOsm of trehalose (76). Trehalose confers a greater cryoprotective capacity to the base extender when added up to 100 mOsm. Other studies have also reached similar conclusions (45, 85). In addition, sperm plasma membrane evaluation by ultramicroscopy indicated better cryoprotective effects on sperm frozen in an extender containing trehalose, there was a significant reduction in the number of damaged membranes (45). In an earlier study by our team, it was found that trehalose had superior cryoprotective effects on sheep sperm’s ultrastructure, compared to other cryoprotectants, such as natural or synthetic ice blockers (70).

Cryopreserving sheep semen in an extender with 100 mM trehalose resulted in a decrease in oxidative stress caused by the freezing and thawing process due to the antioxidant proprieties of trehalose (86). In goats, a tris-based extender supplemented with trehalose at 100 mM can improve post-thaw semen characteristics (87). According to an additional study, adding 150 mM trehalose to the tris-citric acid-egg yolk-fructose diluent resulted in higher efficiency for goat sperm cryopreservation due to its improved motility, viability, plasma membrane integrity, and acrosome integrity (81). Moreover, there are still some disagreements regarding trehalose’s cryoprotective properties for sperm (3, 56). Trehalose and sucrose have comparable abilities to maintain the motility of frozen sheep sperm (54). According to a different study, trehalose does not enhance the quality of frozen goat spermatozoa (35). In addition, trehalose was found to have detrimental effects on the post-thaw kinetic sperm parameters when dimethylacetamide was present (88). Besides, the post-thaw motility and morphology were not improved when the trehalose content in the freezing extender was 50 mM or 100 mM (89).

In some studies, trehalose and other cryoprotectants were used together to enhance the cryoprotective effects of trehalose on frozen sperm. The addition of a combination of oleic acid and trehalose concentrations to a Tris-based extender can improve the post-thaw quality of ram semen (74). Moreover, the combined addition of fetuin and trehalose to the tris-based extenders can lower the overall glycerol usage concentration to 3%, reducing the harmful effects of glycerol and improving the quality of cryopreserved ram sperm (77). Likewise, the post-thaw of ram sperm was improved with 60 mM trehalose (79). Besides, it was observed that there was a positive synergic impact of iodixanol and trehalose on cryosurvival of semen (90). When the combination of antipain (10 μM) and trehalose (30 and 60 mM) was included, they conferred an extraordinary cryosurvival capacity (91). In a previous study, in a soybean lecithin-based extender, a combination of 100 mM trehalose and 5% glycerol was the best combination to realize a better post-thaw quality of ram sperm (80). In goats, supplementation of 200 nM MitoQ alone or along with 150 mM trehalose to semen extender can improve the quality of cryopreserved sperm, which may be related to improved antioxidant enzymatic defense and mitochondrial activity and reduced DNA fragmentation (78). Another investigation suggests that adding 3 mM and 6 mM pentoxifylline or 50 mM and 70 mM trehalose reduces the damage caused by cooling and cryopreservation processes (83). Furthermore, freezing goat sperm in a trehalose-egg yolk extender with a sufficient concentration of sodium dodecyl sulfate greatly increased the sperm’s ability to freeze (92). We can summarize that the effect of trehalose depends on the concentration, extenders, breed, species and cryopreservation protocols. Table 1 summarizes the effects of trehalose, alone or combined with other substances, on semen cryopreservation in small ruminants.

**Table 1:** Effects of trehalose (alone or in combination) on semen parameters in small ruminants during cryopreservation.

Trehalose (alone or with other substances)	Ruminant (sheep or goat)	Doses	Effect on semen cryopreservation	References
Trehalose alone	Sheep	50–100 mOsm	Improved post-thaw sperm motility, viability, and membrane integrity. and reduced oxidative stress.	(45, 76, 86)
Trehalose alone	Goat	100 mM	Improved post-thaw motility, viability, and acrosome integrity. Reduced oxidative stress and enhanced membrane integrity and ultrastructure preservation.	(81, 87)
Trehalose alone	Goat	150 mM	Improved sperm motility, viability, and plasma membrane integrity.	(81)
Trehalose + oleic acid	Sheep	Not specified	Improved post-thaw motility and overall semen quality.	(74)
Trehalose + fetuin	Sheep	Not specified	Reduced glycerol concentration (to 3%), which lessened glycerol’s harmful effects while improving sperm motility and membrane integrity.	(77)
Trehalose + iodixanol	Sheep	60 mM	Enhanced sperm cryosurvival.	(90)
Trehalose + antipain	Sheep	30–60 mM	Increased sperm cryosurvival and membrane integrity.	(91)
Trehalose + glycerol	Sheep	100 mM trehalose +5% glycerol	Best post-thaw sperm quality in terms of motility, viability, and morphology.	(80)
Trehalose + glycerol	Sheep	100 mM trehalose +5% glycerol	Best post-thaw sperm quality in terms of motility, viability, and morphology.	(80)
Trehalose + glycerol	Sheep	100 mM trehalose +5% glycerol	Best post-thaw sperm quality in terms of motility, viability, and morphology.	(80)
Trehalose + MitoQ	Goat	150 mM trehalose +200 nM MitoQ	Improved post-thaw motility, viability, mitochondrial activity, antioxidant defense, and reduced DNA fragmentation.	(78)
Trehalose + pentoxifylline	Goat	50–70 mM	Reduced damage during cryopreservation and cooling processes, improving sperm motility and membrane integrity.	(83)
Trehalose + sodium dodecyl sulfate	Goat	Not specified	Enhanced sperm’s ability to withstand freezing, improving motility and membrane integrity.	(92)
Trehalose (compared with other sugars)	Sheep	100 mM	Comparable motility preservation to sucrose.	(54)

## 4 The role of trehalose as an antioxidant in semen cryopreservation of small ruminant

Trehalose, a non-reducing disaccharide, has gained prominence in cryobiology due to its multifaceted protective properties, particularly as an antioxidant during semen cryopreservation (93, 94). The cryopreservation process induces osmotic stress and causes the generation of excessive reactive oxygen species (ROS), leading to oxidative stress, which is a major cause of sperm damage (95).

Trehalose’s antioxidant effect stems from its ability to scavenge free radicals, thereby preventing ROS accumulation. It stabilizes cell membranes by forming a glass-like structure around phospholipid bilayers during freezing, which preserves the integrity of the sperm membrane against cold shock and osmotic stress (96). Studies have demonstrated that trehalose significantly reduces the levels of malondialdehyde a marker of lipid peroxidation, thereby maintaining the integrity of sperm membrane lipids. Additionally, trehalose prevents mitochondrial dysfunction, which is a key source of endogenous ROS during cryopreservation, ensuring higher post-thaw ATP levels and energy metabolism (78). Furthermore, trehalose has been shown to modulate the activity of antioxidant enzymes such as superoxide dismutase (SOD) and glutathione peroxidase (GPx) during cryopreservation (50). By maintaining these enzymatic defenses, trehalose reduces oxidative damage to sperm DNA and proteins, resulting in improved sperm chromatin integrity and lower levels of DNA fragmentation post-thaw (47). Furthermore, its antioxidant properties protect the sperm membrane against the attacks enacted by free radical to ROS (86). Recently it has been demonstrated that trehalose can protect sperm from oxidative stress by enhancing antioxidant capacity (83). Moreover, lower doses of trehalose reduce lipid peroxidation and protect the spermatozoa (84).

The inclusion of trehalose in cryopreservation extender has been widely reported to enhance antioxidant activity, decrees the oxidative stress and improve the sperm motility and membrane integrity during sperm storage (45, 97).

## 5 The proposed mechanisms of action of trehalose during cryopreservation of semen

Different from monosaccharides including fructose and glucose, trehalose, acting as a sugar, cannot permeate the plasma membrane. As such, its primary role is that of an extracellular cryoprotectant (98, 99). The report by Crowe et al. (100) suggested that trehalose needs to be present on both sides of the plasma membrane in order to have the best protective effects. To address this issue, several technologies have been developed, including microinjection of trehalose into cells, transfection to express trehalose in mammalian cells and thermotropic lipid phase transition (101–104). Research is still needed to determine whether these technologies could introduce trehalose into sperm cryopreservation. Furthermore, the exact mechanism for the effect of trehalose on semen cryopreservation remains unclear.

**Figure 1:**
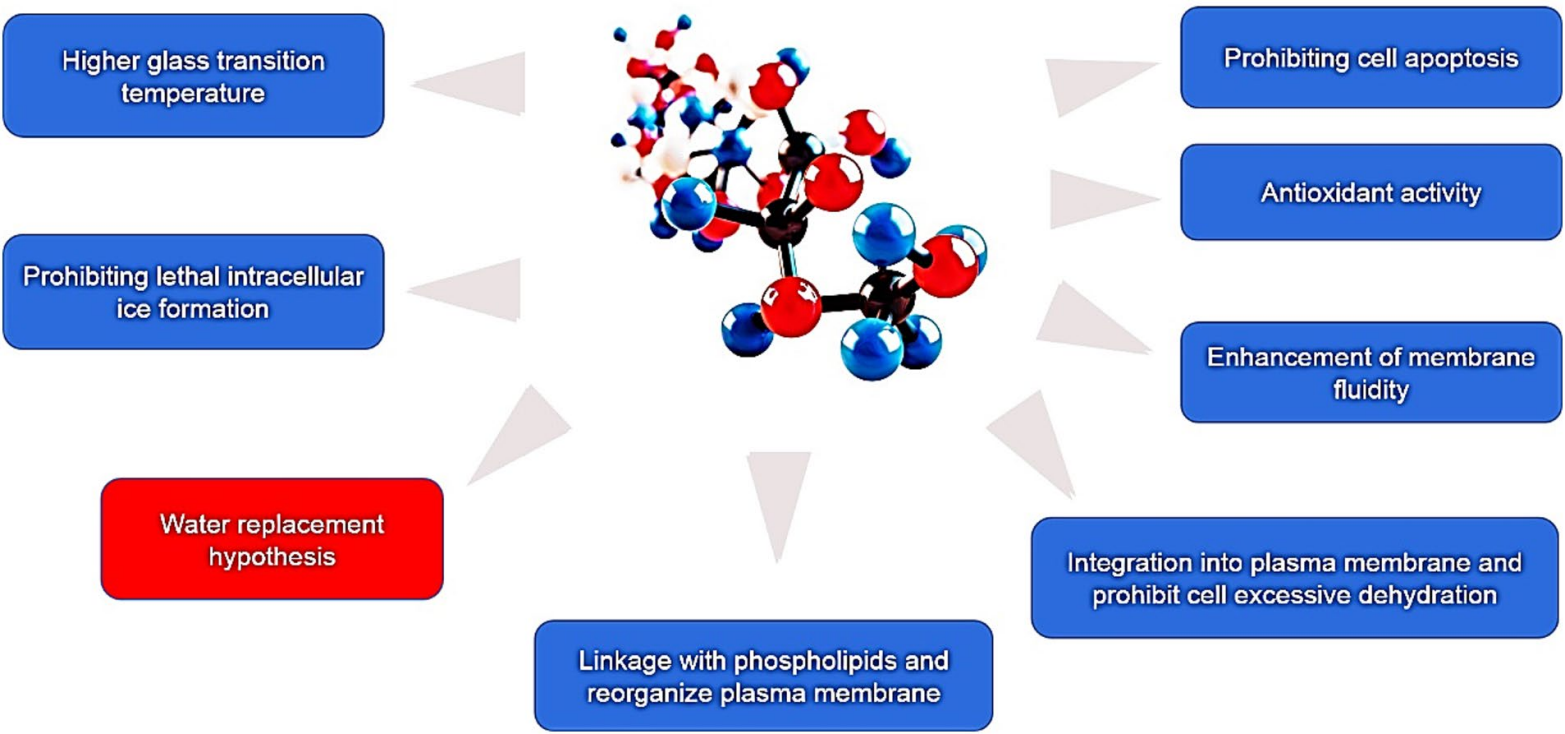
The present cryoprotective hypotheses of trehalose during cryopreservation of mammalian sperm.

**Figure 2:**
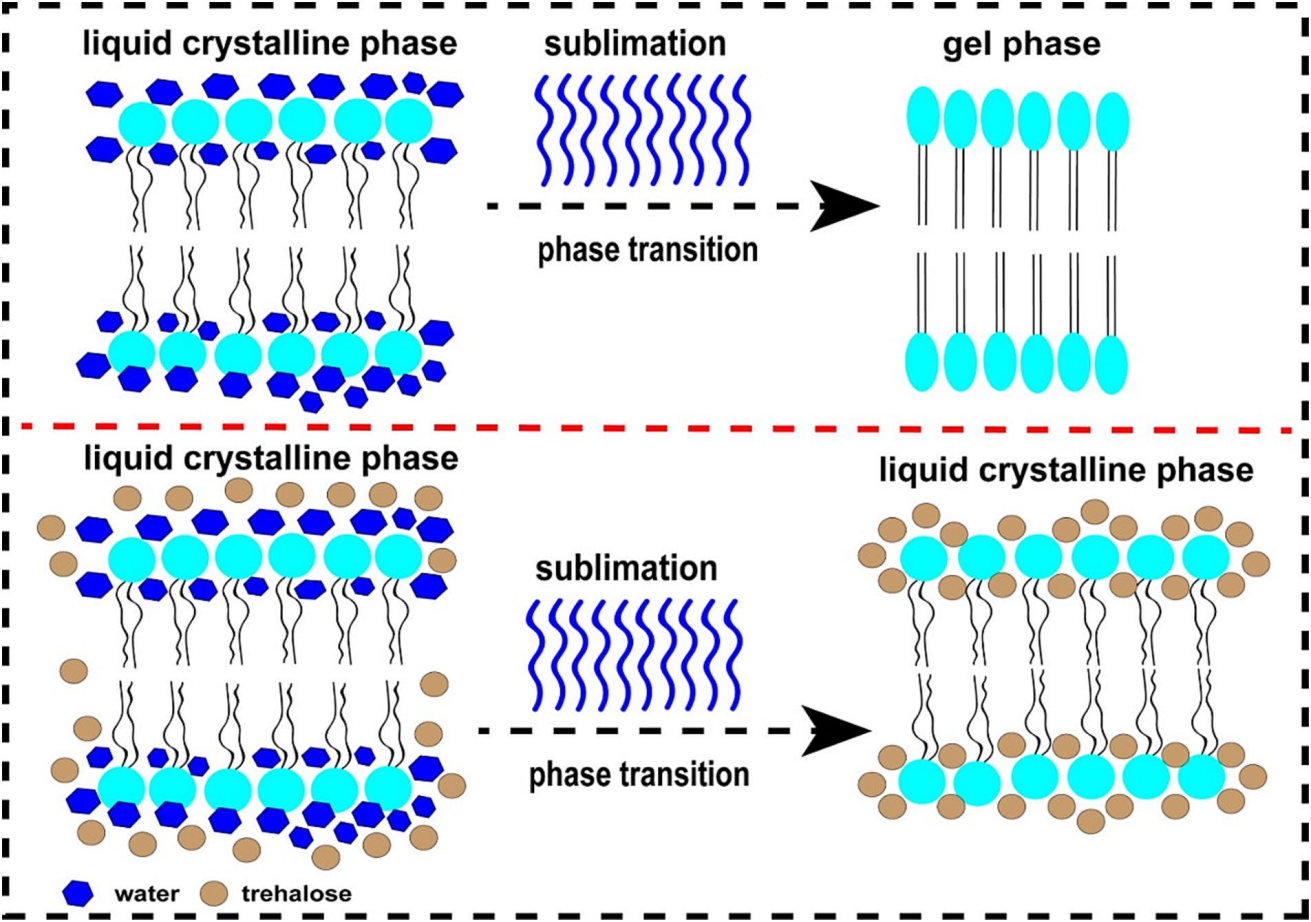
The diagram of water replacement hypothesis. The upper figure represents the changing in sperm plasma membrane from normal liquid crystalline phase to gel phase during freezing in the absence of trehalose. The below figure represents the maintenance of liquid crystalline phase in sperm plasma membrane after freezing in the presence of trehalose.

At present, numerous hypotheses were recommended along with the enhancement of extracellular vitrification formation, prohibiting intracellular ice formation, stabilization of liquid crystalline within the plasma membrane, linkage with phospholipids and reorganization of the plasma membrane, enhancement of membrane fluidity, antioxidant activity, reduced apoptosis, etc. (Figure 1). At first, an excessive glass transition temperature (Tg) is a crucial function of trehalose (105). The Tg of trehalose (−30°C) is a considerably higher than that of different conventional cryoprotectants, which include ethylene glycol (−8°C) and glycerol (−65° C) (106). Therefore, the presence of trehalose in the media may also make contributions to extracellular vitrification formation and decrease in ice crystal production. Secondly, trehalose can increase extracellular osmotic pressure, cause cell dehydration and decrease the formation of lethal intracellular ice (9). Thirdly, trehalose might also additionally update the water shell of macromolecules with the aid of hydrogen bonding and keep away from cryodamage resulting from the freezing and thawing process, in line with the “water replacement hypothesis (Figure 2) (57, 58). Crowe et al. (58) believed that owing to its ability to replace the water shell of macromolecules, trehalose may prevent injury during freezing or drying. As a replacement for the water molecule, trehalose can link membranes or other macromolecules by hydrogen bonding, which is thought to be a necessary condition for reducing the liquid crystalline to gel phase transition temperature (107, 108). Many researchers have mentioned the stabilization mechanism of trehalose in frozen or lyophilized organic cells (109, 110). As a substitute for the water molecule, trehalose can hyperlink membranes or different macromolecules through hydrogen bonding, which may be a notion to be an important circumstance for lowering the liquid crystalline to gel segment transition temperature (58, 111). In addition, the supplementation of trehalose can enhance plasma membrane fluidity of sperm (97). Moreover, trehalose can also link with plasma membrane phospholipids, reorganize plasma membrane, and make sperm survive through the freezing and thawing process (112, 113). It can be integrated into the plasma membrane and prohibit the excessive dehydration of sperm during the cryopreservation process, consequently reducing the physical damage caused by abnormal variations of cell volume (114). In addition, the cryoprotective roles of trehalose can also be related to its antioxidant activity. Finally, in ram, trehalose suppresses lysophosphatidylcholine-precipitated acrosome response in sperm, therefore enhancing cryosurvival of sperm (115).

As of now, the omics innovations technologies have been utilized within the inquiry about small ruminants’ sperm. In understanding with the show reports, the cryopreservation process can modify the abundance of transcripts (116–122), proteins (6, 123–127), metabolites (128–130) and lipids (131). In a few scattered thoughts, about, analysts endeavored to investigate the cryoprotective components of trehalose on little ruminant sperm amid the cryopreservation handle from the viewpoint of transcripts, proteins, and metabolites. In a previous study, the group used electrophoresis technology to assess the relationship between the presence of protein in ram plasma and the quality of semen frozen with 5% glycerol or 100 mM trehalose (132). A total of 26 bands were identified in ram’s seminal plasma. In another study, a total of 1,269 proteins were identified using the isobaric tag for relative and absolute quantification (iTRAQ) strategy combined with parallel reaction monitoring (PRM) Among them, 21 differentially expressed proteins were identified. These proteins were involved in oxidoreductase activity, stress response, and catabolic processes, which may be associated with the cryoprotective effects of trehalose (56). Regarding changes in sperm metabolites after freezing in the presence of trehalose, a research group used GC–MS-based metabolomics to investigate the effects of trehalose on goat sperm (85). 48 different metabolites were found. L-isoleucine, L-leucine, L-threonine, and dihydroxyacetone are synthesized through this pathway, including valine, leucine, and isoleucine biosynthesis, glycerolipid metabolism, and aminoacylRNA biosynthesis (85). For this reason, they thought that trehalose played an important role in changing the amino acid and glycerol metabolism processes in sperm (35). Recently, the use of the RNA sequencing (RNA-Seq) approach to investigate the consequences of the cryopreservation procedure on mammalian sperm transcript profiles became a hot spot (133–135). To the best of our knowledge there are no published reports to elucidate the effects of trehalose on the post-thaw small ruminant sperm based on the changes of sperm-derived RNAs. According to our unpublished research, storage conditions significantly alter the transcription profiles of sheep sperm. However, no mRNA had different expression levels between the control group and the trehalose group. Therefore, we initially hypothesized that the inhibitory effect of trehalose might be unrelated to transcriptomic changes of sperm during storage. However, this result requires further investigation.

## 6 The future application prospects of trehalose in semen cryopreservation

Although there are still ongoing debates, most studies support the positive effect of trehalose on small ruminant spermatozoa during storage (45, 47, 79). It should be noted that the cryoprotective effect of trehalose may depend on certain factors such as the species, breed, and composition of the extenders used (3). Moreover, the mechanism underlying this protective function is not yet fully clear. According to current studies, the use of omics technologies, including genomics, transcriptomics, proteomics and metabolomics, may be the best solution to investigate the mechanism of trehalose utilization. In particular, changes in the structure of RNA molecules such as mRNA, lncRNA, circRNA and microRNA may be an important mechanism explaining the functional role of trehalose (133, 136–139). Semen quality may also be associated with sperm fertility (102, 140, 141) and may give us some clues about the role of trehalose. However, the limitations of this study are related to how we can obtain useful information from already published studies, since most of the current studies on spermatozoa research include basic bioinformatic analysis. Nevertheless, some results are completely speculative and do not stand up to scrutiny. However, according to the “water exchange hypothesis” theory, most of the major trehalose must enter the cell to be effective in the inhibition process (57, 58). Finally, the mechanism of trehalose loading in yeast is complex. The actual effect of trehalose on sperm quality remains to be confirmed.

## 7 Conclusion

Trehalose has demonstrated beneficial effects during cryopreservation of small ruminant sperm according to the most current investigations. However, some disputes about the effects of trehalose on sperm characteristics after the cryopreservation process still exist. Currently, some hypotheses, such as the water replacement hypothesis, enhancement of membrane fluidity, and prevention of ice formation, have been proposed to explain the possible functional roles of trehalose during semen cryopreservation. However, the real cryoprotective mechanism of trehalose still needs to be determined.

At present, the rapid development of omics technologies including transcriptomics, proteomics, and metabolomics may provide new opportunities for elucidating the cryoprotective roles of trehalose. In addition, according to the “water replacement hypothesis,” trehalose should be present in cells to ensure its optimally protective effects on frozen cells. However, how to load trehalose into sperm is a difficult task that may influence the actual action effects of trehalose. Finally, the cryoprotective function of trehalose on sperm still needs verification by artificial insemination in the field.
